# Chronic spontaneous non-aneurysmal aortic rupture treated with endovascular surgery

**DOI:** 10.31744/einstein_journal/2024RC1113

**Published:** 2024-11-19

**Authors:** Bruno Jeronimo Ponte, Viviane Galli Dib, Andressa Cristina Sposato Louzada, Júlia Freire Castanheiras de Paiva Ferreira, Lucas Lembrança Pinheiro, Cynthia de Almeida Mendes, Nelson Wolosker

**Affiliations:** 1 Hospital Israelita Albert Einstein São Paulo SP Brazil Hospital Israelita Albert Einstein, São Paulo, SP, Brazil.; 2 Hospital Israelita Albert Einstein Faculdade Israelita de Ciências da Saúde Albert Einstein São Paulo SP Brazil Faculdade Israelita de Ciências da Saúde Albert Einstein, Hospital Israelita Albert Einstein, São Paulo, SP, Brazil.; 3 Universidade de São Paulo Faculdade de Medicina São Paulo SP Brazil Faculdade de Medicina, Universidade de São Paulo, São Paulo, SP, Brazil.

**Keywords:** Rupture, spontaneous, Aneurysm, false, Patient discharge, Penetrating atherosclerotic ulcer, Aorta, abdominal, Aneurysm, Prostheses and implants, Angiography, Endovascular procedures, Tomography, X-ray computed, Magnetic resonance imaging, Aged, 80 and over, Intensive care units

## Abstract

Spontaneous non-aneurysmal aortic rupture is rare and is usually attributed to penetrating aortic ulcers, infections, tumor infiltrations, or inflammatory and collagen diseases. Chronic rupture is infrequent but extremely rare in non-aneurysmal aortas, which makes diagnosis difficult because the absence of an aneurysm can mislead the physician to rule out rupture. Here, we describe the case of an 85-year-old male, who was undergoing oncological investigation for weight loss, inappetence, and back pain. Computed tomography and magnetic resonance imaging performed 3 months before admission showed a contained pseudoaneurysm of the infrarenal aorta associated with significant aortoiliac calcification and images suggestive of peritoneal implants. The patient was referred to our oncological center and underwent abdominal computed tomography for oncological investigation and staging. The patient was urgently admitted to the intensive care unit after a critical finding of contained rupture of the infrarenal aorta during the scan. Endovascular repair was indicated, and the patient was successfully treated with implantation of an Endurant IIs 25 × 25 × 70 mm endoprosthesis. No procedural complications were observed. Postoperative course was uneventful, and the patient was discharged on the fifth postoperative day. Control computed tomography performed 1 and 6 months after surgery showed no leaks. This case emphasizes the importance of communication between the radiologists and attending physicians. As the rupture was punctual and well defined in the computed tomography and angiography images, endovascular repair with an aortic cuff was safely performed, and the procedure time was reduced.

## INTRODUCTION

Spontaneous non-aneurysmal aortic rupture is rare and usually attributed to penetrating aortic ulcers (PAUs),^(1)^ infections,^(2)^ tumor infiltrations, or inflammatory and collagen diseases.^(3,4)^

Chronic aortic rupture is also infrequent, usually associated with aneurysmal aortas, and can occur when the retroperitoneal location of the perforation allows for tamponade.^(5)^

The association between chronic and spontaneous non-aneurysmal aortic ruptures is extremely rare. To the best of our knowledge, there is only one report in the literature but this occurred in a non-native aorta that was an aortic graft without pseudoaneurysms, treated with an extra-anatomical bypass.^(6)^

Herein, we report a case of chronic spontaneous non-aneurysmal infrarenal aortic rupture that was successfully treated with endovascular repair (EVAR).

## CASE REPORT

An 85-year-old male was admitted to our hospital with critical findings of non-aneurysmal aortic rupture during an elective abdominal computed tomography (CT) scan.

The patient had a history of hypertension and hypothyroidism. He had been treated for colon cancer 25 years previously with surgery and adjuvant chemotherapy. In addition, he had undergone radical prostatectomy for prostate cancer 20 years previously. He was under oncological investigation at another hospital due to a 10kg weight loss in 9 months and back pain. Computed tomography and magnetic resonance imaging performed in July 2022 showed a pseudoaneurysm of the infrarenal aorta associated with significant aortoiliac calcification and images suggestive of peritoneal implants.

Despite the critical aortic finding, the patient was not referred to the emergency department (ED), but was referred on an outpatient basis in our cancer center, where the vascular team was called in by the radiology team immediately after the CT scan.

Upon admission in October 2022, the patient complained of mild chronic back pain for the past 5 months, with no acute worsening. The patient denied experiencing trauma, abdominal pain, or other critical events during the study period.

On physical examination, he was in good general condition, hemodynamically stable (blood pressure 147×72mmHg; heart rate, 56bpm; StO2 on room air, 95%), lucid, and oriented. The patient presented with an infraumbilical scar without any abnormalities, painless abdominal palpation, and a painless pulsatile epigastric mass. The extremities were well perfused and all pulses were symmetrical.

Laboratory findings showed a hemoglobin level of 10.3g/dL, platelet count of 189,000µL, and creatinine level of 1.23mg/ dL (clearance, 53mL/min).

**Figure 1:**
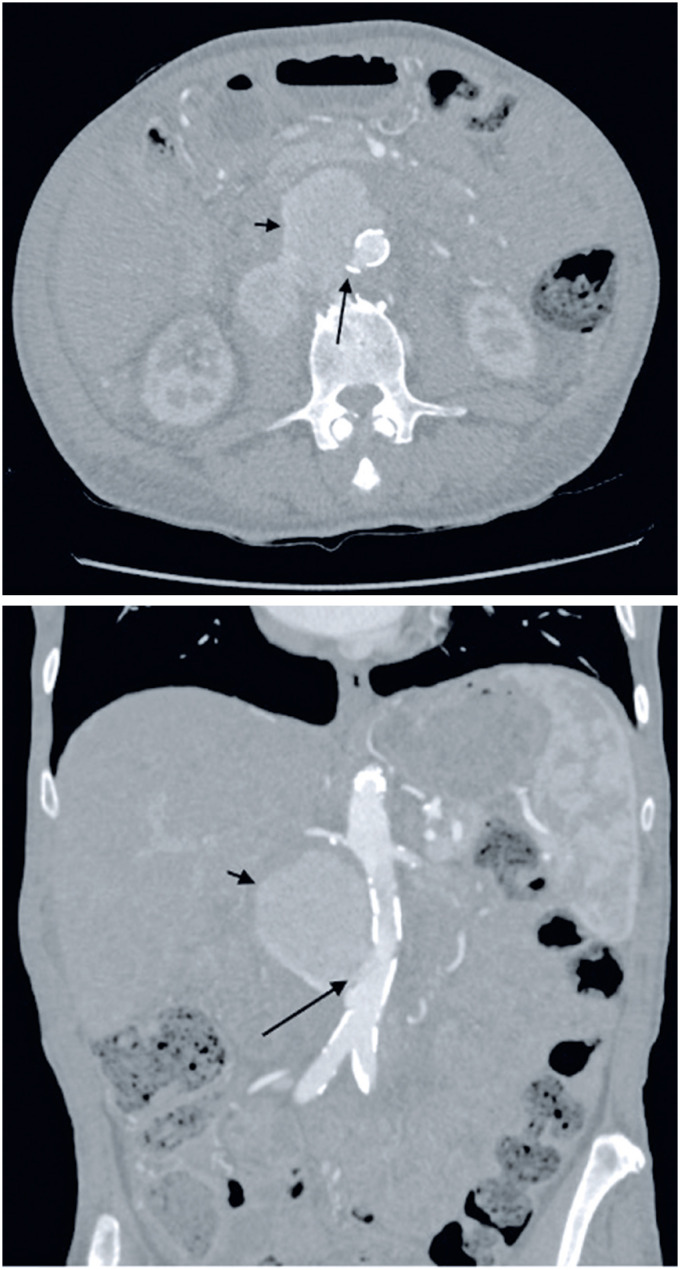
Contained aortic rupture. Arrows: wall rupture; Short arrows: bleeding contained along the retroperitoneum

During the arterial phase, the patient's CT scan demonstrated no aneurysms, intense atherosclerosis, and a discontinuity of the aortic wall 5.1cm below the left renal artery (the lowest one) and 2.7cm above the aortic bifurcation, with extravasation of contrast into the retroperitoneum (Figure 1).

Given the rupture, favorable anatomy, and patient's comorbidities (advanced age, previous abdominal surgery, and suspected peritoneal carcinomatosis), emergency EVAR was indicated; however, under general anesthesia, a chronic and stable condition was considered.

**Figure 2:**
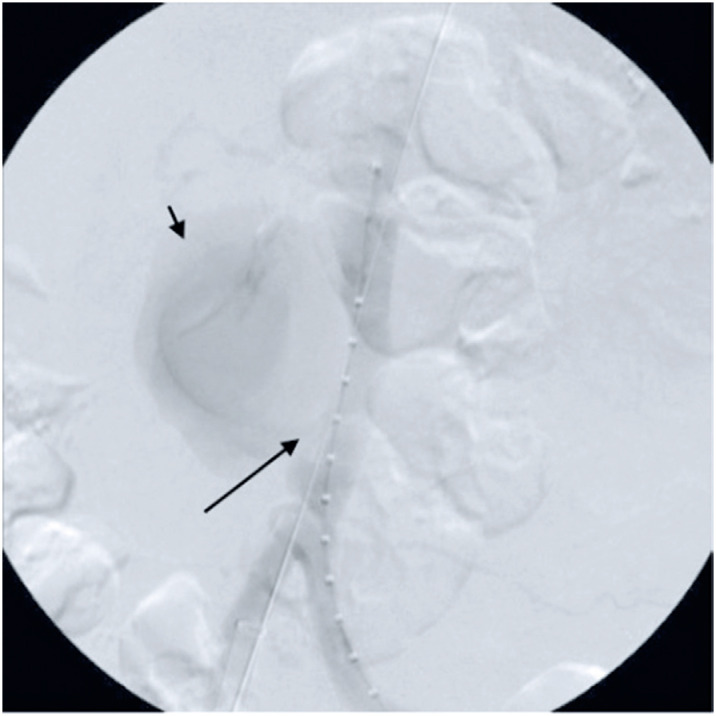
Initial aortography. Long arrow: rupture of the aortic wall; Short arrow: tamponade bleeding

**Figure 3:**
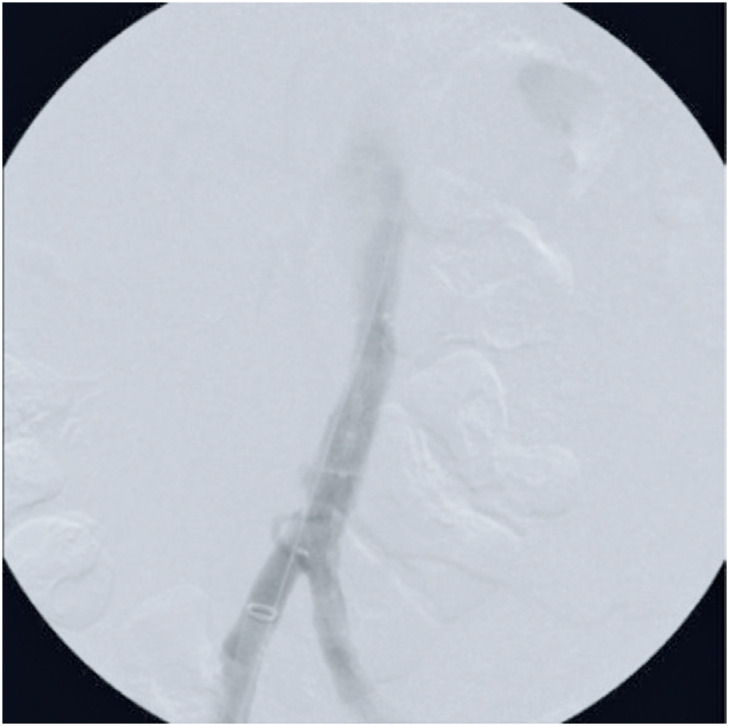
Final aortography. Treatment of the rupture with endoprothesis placement, demonstrating bleeding control

Intraoperative aortography revealed an aortic rupture with contrast leakage and another bulging image compatible with an aortic ulcer 1cm above the aortic bifurcation (Figure 2). An Endurant IIs 25 × 25 × 70mm stent graft (Medtronic, Minnesota, US) was implanted immediately above the bifurcation, with complete resolution of the rupture and aortic ulcer and no evidence of leaks at the end of the procedure (Figure 3).

The patient received 2,000mL of crystalloids and a bag of concentrated red blood cells; 58mL of iodinated contrast was administered, and the total diuresis was 300mL (1.03mL/kg/h).

No surgical or anesthetic complications occurred. The patient was extubated in the operating room, and no vasoactive drugs were required after the surgery.

Patient was transferred to the intensive care unit stable, in good general condition. The postoperative course was uneventful and uneventful. On physical examination, abdominal palpation remained painless, the mass was no longer pulsatile, and there were no complications related to the inguinotomy or arterial puncture. On the first postoperative day (POD), food intake was initiated with good acceptance. On the second POD, the patient had normal diuresis and bowel habits, and was able to deambulate. On the fourth POD, he was discharged in good general condition with no pain.

**Figure 4:**
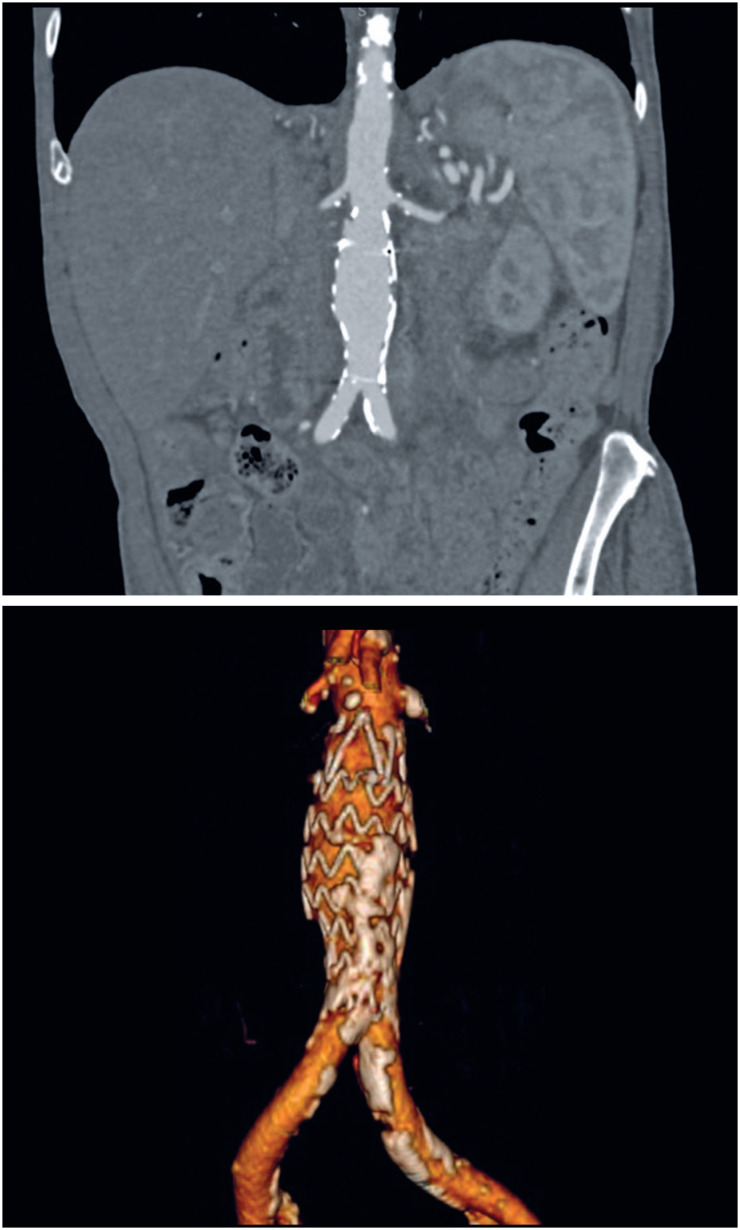
Computed tomography performed 6 months after surgery. Total exclusion of the pseudoaneurysm and absence of endoleaks. No migration of the endoprosthesis is demonstrated

Postoperative CT revealed no signs of leakage or other complications (Figure 4).

This study was approved by the Ethics Committee of the *Hospital Israelita Albert Einstein* (CAAE: 75777623.8.0000.0071; #6.600.514) on December 23, 2023. The patient was invited to participate in the study, and a consent form was signed for the case report and the use of images.

## DISCUSSION

We present the first case of chronic spontaneous rupture of a non-aneurysmal infrarenal native aorta, an extremely rare but potentially fatal condition.

The etiology of the rupture remains uncertain, as EVAR did not allow us to obtain a sample for anatomic-pathological analysis. Nonetheless, considering the aortic calcification and bulging that appeared on aortography, the most likely underlying cause of rupture was a PAU.

A PAU is defined as a focal ulcerative lesion that develops in the region of an atheromatous plaque penetrating the internal elastic lamina of the vascular wall.^(7)^ It is frequently observed in the thoracic and abdominal aortas of patients with severe atherosclerosis.^(7)^It has a low prevalence, and its natural history and optimal management remain controversial.^(8)^ Nonetheless, every patient with a PAU should be referred to a vascular service. When asymptomatic, PAU may be managed conservatively with close follow-up.^(7,9)^ But when complicated, surgery must be indicated, with EVAR being the gold standard.^(7,9,10)^ In our case, the probable PAU had already been aggravated by the most lethal complication, aortic rupture.

When the rupture is blocked and the patient is stable and oligosymptomatic, diagnosis can be challenging, especially in the absence of an aneurysm.^(5)^However, it is noteworthy that the patient underwent two scans with this finding over the course of 3 months and was not referred to the ED. We believe that this arc of communication between radiologists and assistant physicians to report such critical findings is crucial for saving lives and should be mandatory in all services.

Fortunately, our patient was treated in a timely manner, and EVAR with implantation of a cuff instead of a standard aortobiiliac endoprosthesis was effective and reduced the procedural time.^(1)^

## CONCLUSION

Chronic and spontaneous non-aneurysmal aortic rupture is rare, and its diagnosis may be challenging. Therefore, it is imperative to establish a communication arc between radiologists and assistant physicians. Discontinuity of the wall can be successfully treated with an endovascular cuff, thereby reducing the procedural time.
